# Recovery of monosaccharides from dilute acid corncob hydrolysate by nanofiltration: modeling and optimization

**DOI:** 10.1039/c8ra00236c

**Published:** 2018-04-03

**Authors:** Kangkang Jiang, Han Kuang, Taotao Qin, Mingkai Song, Jingwei Zhou, Pengpeng Yang, Wei Zhuang, Hanjie Ying, Jinglan Wu

**Affiliations:** College of Biotechnology and Pharmaceutical Engineering, Nanjing Tech University Xin Mofan Road 5 Nanjing 210009 China wujinglan@njtech.edu.cn +86-25-58139389 +86-25-86990001; National Engineering Technique Research Center for Biotechnology Nanjing China; Jiangsu National Synergetic Innovation Center for Advanced Materials Nanjing China; State Key Laboratory of Materials-Oriented Chemical Engineering Nanjing China

## Abstract

In this work nanofiltration technology has been employed for removal of inhibitors and recovery of monosaccharides from dilute acid lignocellulose hydrolysates. The influences of feed solution pH, permeate flux, and Na_2_SO_4_ concentration on the rejection of monosaccharides and inhibitors were investigated. The results showed that the pH for the separation of carboxylic acids and furans from monosaccharides should be as low as possible. With increase of Na_2_SO_4_ concentration carboxylic acid and furan rejection decreased. Subsequently, the Donnan steric pore and dielectric exclusion model coupled with mass balance was used to predict the rejection of solutes at different permeate fluxes. In order to select a suitable permeate flux and operating time, multi-objective optimization was carried out to obtain the maximum total inhibitor removal efficiency, the maximum monosaccharide recovery rate, and the minimum water consumption. The optimal operating conditions were then verified using the real hydrolysate as feed solutions. More specifically, for the treatment of 6 L of a hydrolysate solution, 13 L of water and a treatment time of 35 min were required. This process allowed the removal of 90% inhibitors, while 93.55% glucose, 90.75% xylose, and 90.53% arabinose were recovered. Finally, a batch column equipped with a strong acid cation exchange resin was employed to recover the monosaccharides from the hydrolysate. Using water as an eluent, 95.37% of the sulfuric acid and 94.87% of the monosaccharides were recovered. In all, we demonstrated that the combination of nanofiltration with electrolyte exclusion chromatography is a promising integrated process for the recovery of monosaccharides and inorganic acids from dilute acid corncob hydrolysates.

## Abstract 2

In this work Multi-objective optimization has been employed to obtain the optimal permeate flux (*j*_v_) and operating time (*t*) during the diananofiltration detoxification of dilute acid corncob hydrolysate process.

## Graphical abstract

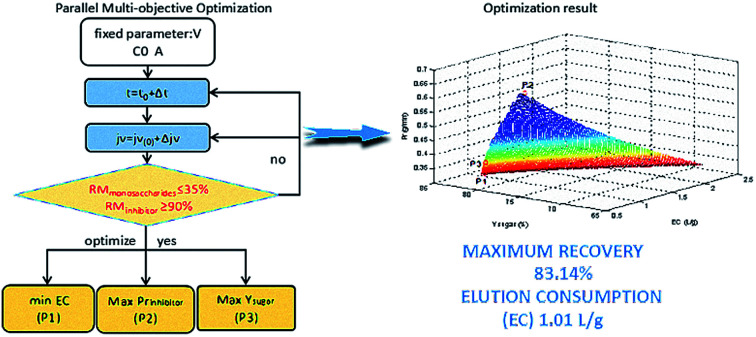

## 1. Introduction

Lignocellulosic biomass, such as woody materials and agricultural residues, is an abundant, readily available, and renewable feedstock for the production of biofuel. However, the utilization of such biomass generally requires pre-treatment processes, through which polymeric carbohydrates are decomposed to monosaccharides.^1^ To date, a number of pretreatment methods have been proposed, including enzyme catalysis, hot water pretreatment, thermal pretreatment with mineral acids, or alkaline treatment,^2^ with dilute acid pretreatment being the most commonly used method.^3^ However, the dilute acid pretreatment method produces a number of by-products, such as furfurals, hydroxymethyl furfural (HMF), phenolic compounds, and acids (*e.g.*, acetic and formic acid),^4,5^ with the presence of such by-products during sugar fermentation being reported to seriously inhibit bacterial growth and the production of the desired bio-based products. For example, even low concentrations of phenolics are lethal to *Clostridium*, which is a widely used bacterium in the production of butanol and butyric acid.^5^ The removal of these inhibitors from hydrolysates is therefore of particular importance.

To date, a number of techniques have been employed for hydrolysate detoxification, including evaporation, activated charcoal adsorption, overliming, neutralization, ion exchange, enzyme treatment, and electrodialysis.^6–8^ However, as expected, these methods exhibit a number of different advantages and disadvantages. For example, overliming produces large quantities of gypsum during the neutralization and detoxification process, while evaporation increases the concentration of non-volatile compounds despite removing volatile compounds. In addition, electrodialysis removes only the compounds that can be dissociated (*e.g.*, *p*-coumaric acid, ferulic acid, syringaldehyde, and vanillin).^9^ In the context of the various fermentation inhibitors mentioned above, furfural, HMF, and phenolic compounds can be removed by adsorption due to their hydrophobic properties, while the separation of acetic acid and formic acid from glucose and xylose is more problematic.

Nanofiltration (NF) is an efficient membrane separation technology that exhibits low energy consumption and unique separation properties. As such, Weng *et al.*^10^ investigated the separation of furans and carboxylic acids from sugars in dilute acid rice straw hydrolysates using Desal-5 Dk nanofiltration, which had a molecular weight cutoff of 150–300 Da. Using a pH of 2.9 and an applied pressure of 24.5–34.3 bar, they achieved maximum separation factors of acetic acid and HMF over xylose of 49 and 43, respectively. In addition, Brás and Guerra *et al.*^11^ employed diananofiltration mode to detoxify hemicellulosic hydrolysates from extracted olive pomace, and reported 99% removal of furans, acetic acid, and formic acid, but a monosaccharide loss of 40%. Furthermore, Maiti *et al.*^12^ used the Donnan steric pore model (DSPM) to characterize the membrane and membrane transport. They concentrated a rice straw acid hydrolysate using a volume concentration ratio of 4, and increased the concentrations of xylose, glucose, arabinose, cellobiose, and inhibitors by 100, 104, 93, 151, and 3%, respectively. However, previous studies have ignored the existence of dilute sulfuric acid, which can have a significant influence on the separation performance of the nanofiltration membrane. Optimization of the operating conditions would therefore be expected to minimize the monosaccharide removal rate and the consumption of water. Moreover, separation of the acid–sugar mixtures produced from the treatment of hydrolysates by nanofiltration could be simplified if sulfuric acid could be recycled.

In this context, electrolyte exclusion chromatography, which is an efficient method for the separation of strong electrolytes from weak electrolytes and nonelectrolytes,^13^ has recently been applied in the fractionation of acid–sugar mixtures. During this process, strong electrolytes are excluded from the strong ion exchange resins either completely or partially due to electrical repulsion caused by the fixed ionic groups in the resin.^14^ In addition, the strong electrolytes break through the resin bed at the interstitial volume due to complete exclusion at infinite dilution. Weak electrolytes and nonelectrolytes are unaffected by the electrolyte exclusion and so propagate through the column slower than strong electrolytes. Thus, Sun *et al.*^15^ used a Dowex 1X8 column to separate sulfuric acid and sugars in concentrated sulfuric acid hydrolysates of bamboo, and reported sulfuric acid, glucose, and xylose recoveries of 90.5–93.4, 94.9–99.7, and 82.8–88.3%, respectively. In addition, Heinonen and Sainio^16,17^ investigated the recovery of monosaccharides and sulfuric acid from the concentrated acid hydrolysate of lignocellulosic biomass, while Xie *et al.*^18^ employed the electrolyte exclusion chromatography technique for the separation of monosaccharides from dilute acid lignocellulosic hydrolysates. Furthermore, Springfield and Hester^19^ investigated the fractionation of a solution containing sulfuric acid (10 wt%) and glucose (10 wt%) using a four-zone simulated moving bed for binary separations. These results suggest that the recovery of sulfuric acid can indeed be achieved in a number of systems.

Thus, we herein report the coupling of NF and electrolyte exclusion chromatography to remove inhibitors and recover monosaccharides from a dilute acid corncob hydrolysate. The effects of different operating conditions (*i.e.*, flux, pH, and Na_2_SO_4_ concentration) on the separation of acetic acid, formic acid, and furans from monosaccharides are examined, and coupling of the DSPM-DE with mass balance calculations will be employed to predict the rejection of solutes at different permeate fluxes and to simulate the diananofiltration process. To select a suitable permeate flux (*j*_v_) and operating time (*t*) for the diananofiltration process, multi-objective optimization was carried out to obtain the maximum total inhibitor removal efficiency (Pr_inhibitor_), the maximum monosaccharide recovery rate (*Y*_sugar_), and the minimum water consumption (EC). An authentic hydrolysate sample will also be employed to verify the optimized conditions. Following NF, recovery of the monosaccharides and sulfuric acid present in the retentate are attempted using a strong acid cation-exchange resin (PS-DVB) in a batch column.

## 2. Materials and methods

### 2.1. Raw materials and pretreatment

Glucose, xylose and arabinose were purchased from Shanghai Sangon Biological Engineering Co. Ltd. Acetic acid was purchased from Shanghai Shen Bo Chemical Co. Ltd. Ferulic acids, vanillin, HMF, furfural, vanillic acids, formic acids and acetic acids were purchased from Aladdin Reagent Co., Ltd. Corncob was collected from Jingzhou, Hubei Province, China. The strong acid styrene-*co*-divinylbenzene cation-exchange resins Sa-2 was purchase from AnHui Sanxing Resin technology Co. Ltd. Synthetic solutions were prepared in de-ionized water. Solution pH was adjusted to 3, 5, 7 and 9 by addition of HCl/NaOH solutions. Hydrolysate sample was prepared by hydrolyzing corncob (20%, w/v) with 2% H_2_SO_4_ for 150 min in an autoclave at 125 °C. After pretreatment, the liquid fractions were separated *via* vacuum filtration and were stored at 4 °C. Before nanofiltration, hydrolysate was prefiltered with a filter of 0.45 μm.

### 2.2. Membrane and nanofiltration module

**Fig. 1:**
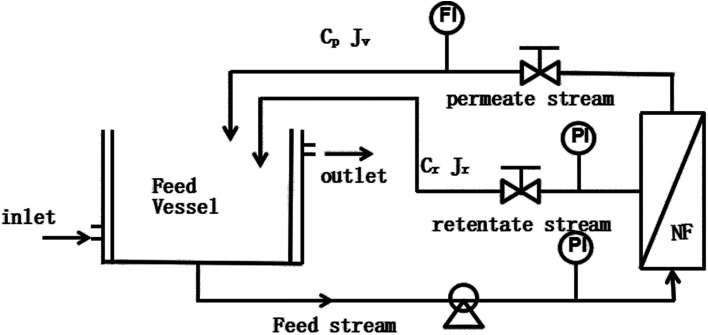
Experimental setup for the nanofiltration (NF) process. PI and FI are the pressure and flow rate indicators, respectively.

A commercial membrane, DK1812-34D (GE Company, USA), was used in this work which has been proven to have high rejection for monosaccharide.^11^ From the information given by the manufacturer, the MWCO of the membrane are 150–300 Da. The effective filtration area of the membrane is 0.32 m^2^. The experimental module is purchased from Sundar Membrane Technology Co. Ltd. The nanofiltration experimental setup used in this work is shown in Fig. 1, which has a feed tank, diaphragm pump, pressure gauge, membrane module, and pressure control valve.

### 2.3. Filtration experiments

Before the experiments were conducted, the membrane was washed with deionized water for several times. Pure water flux of the membrane was measured while the operating pressure varied from 6.0 bars to 24.0 bars. The permeability was then calculated as the slope of the pure water flux *versus* the operating pressure. All filtration experiments were performed in batch mode with the retentate and permeate fully recycled to the feed tank. The temperature was controlled to 25 °C by circulating water into the jacket of the feed tank using a constant-temperature device. Feed and permeate samples were collected for each experimental conditions. The permeate flux *J*_v_ was measured at each operating pressure and calculated using eqn (1).1
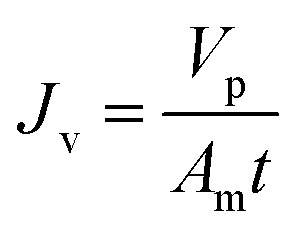
where *V*_p_ is the volume of permeation, *t* is the time, and *A*_m_ is the effective membrane area. Rejection of glucose and xylose were performed at different pH to estimate the pore size. Rejection of Na_2_SO_4_ was performed at varied concentrations to estimate the effective charge density.

### 2.4. Concentration-diananofiltration experiment

The membrane separation process was operated at a concentration-diananofiltration mode. The optimal *J*_v_ and *t* were determined by Parallel Multi-objective Optimization. In the concentration process, 6 L of model solution and hydrolysate were concentrated to 3 L. In the diananofiltration procedure, the permeate flow was continuously removed and equivalent volume of deionized water was added into the feed tank to keep the feed volume constant along the experiment. The samples were collected every 3 minutes.

### 2.5. Column experiments

The monosaccharides separation from sulphuric acid was performed in a batch column. The strong acid PS-DVB cation-exchange resins (gel type) in H^+^ form were used. The resin bed volume is 425 cm^3^ and the bed height was 55 cm. The hydrolysate treated after the nanofiltration was fed in the column. The injection volume was 10 vol% of the resin bed volume. Water was pumped with a constant flow rate of 1 mL min^−1^ through the column. Samples were collected by an automatic collector.

### 2.6. Sample analyses

The concentrations of the monosaccharides, acetic acid and formic acid were measured by an on-line HP Agilent 1100 HPLC system equipped with a RID detector and a Bio-Rad Aminex HPX-87H column. The HPLC analyses were conducted at 55 °C with injection volume of 10 μL. The 0.005 M H_2_SO_4_ was used as an eluent. The concentration of 5-HMF, furfural and phenolic compounds were determined by an Agilent 1100 HPLC with a diode array detector working at 280 nm. The separation was carried out through a Zorbax XDB-C18 column at the temperature of 55 °C. The mobile phase were 0.3% acetic acid (70%) and methanol (30%) mixture at a flow rate of 1.0 mL min^−1^. Sulfuric acid concentrations were calculated by LeiCi DDBJ-350 conductivity meter with a DJS-1CF probe.

## 3. Modeling and calculations

The real (*R*_*i*,real_) and the observed (*R*_*i*,obs_) rejection of solutes represent the separation performance of the nanofiltration membrane, which are defined as eqn (2) and (3)2
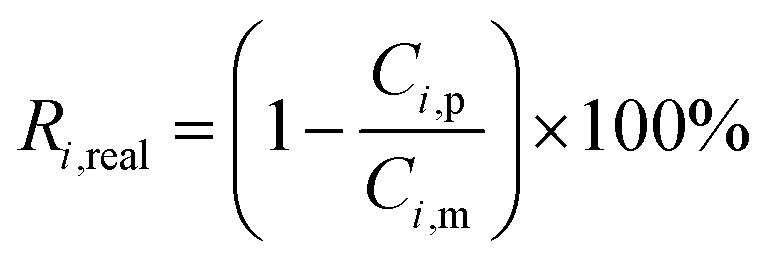
3
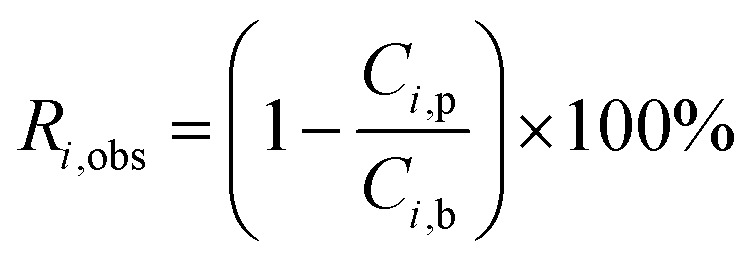
where *C*_*i*,p_ is the concentration of solutes in the permeate, *C*_*i*,m_ is the concentration near the membrane surface, which is difficult to be measured, and *C*_*i*,b_ is the bulk concentration of solutes. Due to the concentration polarization, the bulk concentration is lower than the concentration near the membrane surface. Thus the following correlation of *C*_*i*,b_ and *C*_*i*,m_ is used to obtain *C*_*i*,m_.4
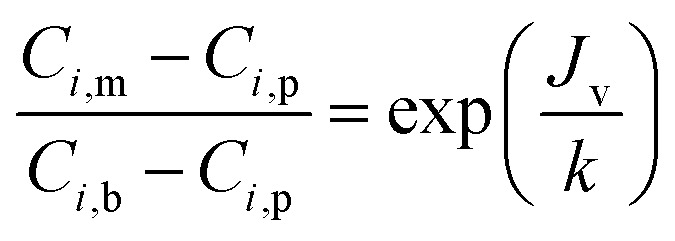


Substitute eqn (2) and (3) for eqn (4),5
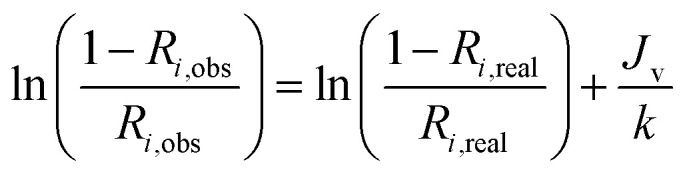
where *K* is the mass transfer coefficient. It can be calculated from eqn (6) in which *d*_c_ is the hydrodynamic diameter, *D* represent diffusivity coefficient, Re is Reynolds number and Sc is Schmidt number.6
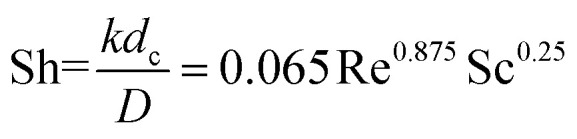


### 3.1. DSPM-DE model

The Donnan-steric-pore-dielectric-exclusion (DSPM-DE) model,^20,21^ which was derived from the extended Nernst–Planck equation, was used in this work to simulate the NF process. The equation can be expressed as:7
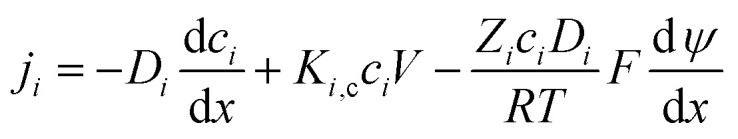


For the uncharged solutes (like the xylose), the electrical potential gradient can be ignored. So the rejection of the solutes can be expressed as:8
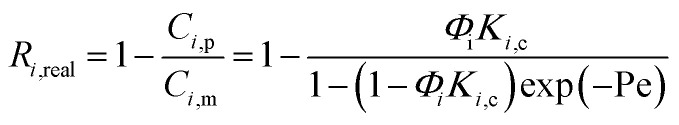
in which *Φ*_*i*_, *K*_*i*,c_ and Pe are the model parameters. Pe can be obtained from eqn (9)9
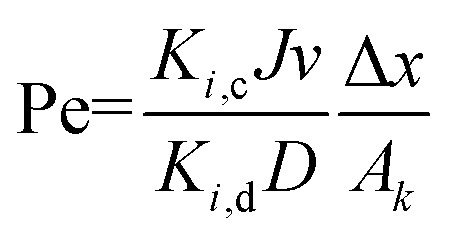
and

10 *K*_*i*,d_ = 1 − 2.3*λ*_*i*_ + 1.154*λ*_*i*_^2^ + 0.224*λ*_*i*_^3^

11 *K*_*i*,c_ = (2 − *Φ*_*i*_)(1 + 0.054*λ* − 0.988*λ*^2^ + 0.441*λ*^2^)

12
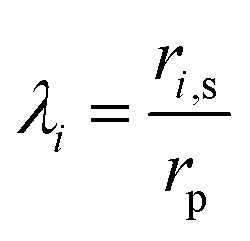

13 *Φ*_*i*_ = (1− λi)

14
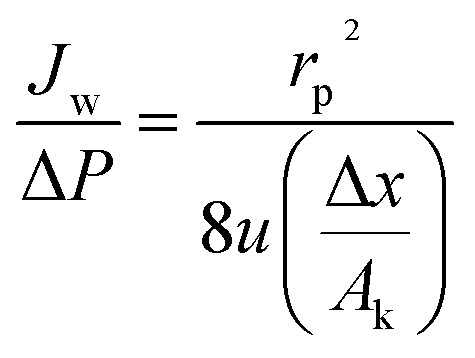
where *r*_*i*,s_ is Stokes radius of solute, *r*_p_ is average membrane pore radius, *J*_w_ is the pure water flux and Δ*P* is the transmembrane pressure. All the model parameters were determined in Section 4.1 in details.

For the charged solutes, the concentration gradient and potential gradient can be expressed as eqn (15) and (16) , respectively.15
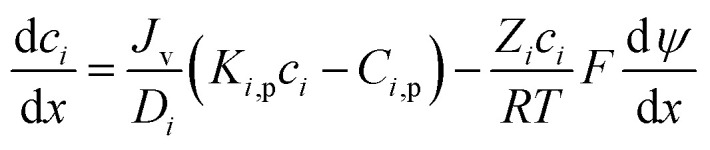
16
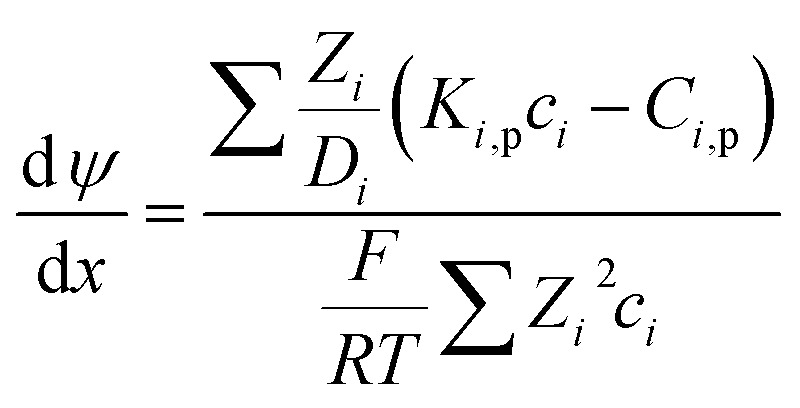


The electroneutrality conditions should be fulfilled:

17 ∑*Z*_*i*_*C*_*i*,b_ = 0

18 ∑*Z*_*i*_*c*_*i*_ + *X*_d_ = 0

where *X*_d_ is effective membrane volume charge density, *Z*_*i*_ is valence of ion i.

In order to solve the above-mentioned ordinary differential equations, the boundary conditions should be included which can be obtained from the Donnan steric equilibrium partition coupled dielectric exclusion effect.19
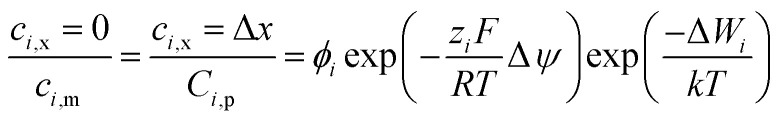


*ϕ*_*i*_ is steric partitioning coefficient, Δ*ψ* is Donnan potential difference and Δ*W*_*i*_ is Born solvation energy barrier. The Δ*W*_*i*_ can be calculated based on the method proposed by Bowen and Welfoot:^22^20
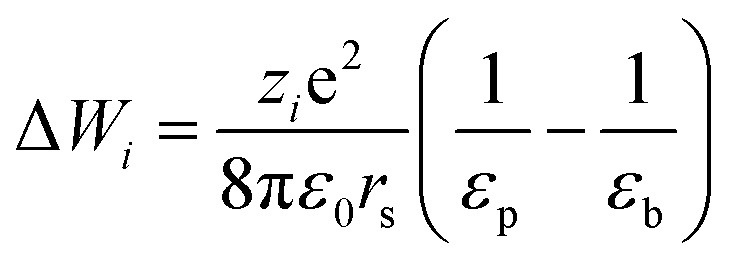


*ε*_p_ and *ε*_b_ are the pore and bulk dielectric constant, respectively. The variation of the average pore dielectric constant was estimated as proposed by Bowen and Welfoot^22^ as follows:21
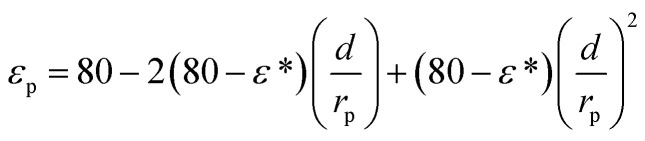
Among which ε* = 6, *ε*_b_ = 80, *d* = 0.28 nm.

### 3.2 Mass balance equations in diananofiltration

The diananofiltration procedure is a batch-continuous process. The solution flux *J*_v_ and volume of feed solution keep constant. The mass balance of the diananofiltration process can be expressed as eqn (22) which has been adopted by Brás and Guerra.^11^22
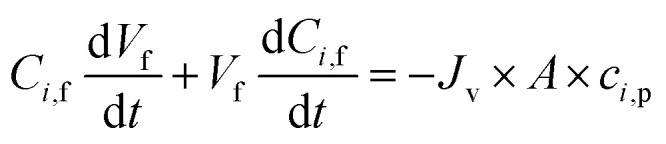


Solve the eqn (22) the concentration of solutes at any time (*C*_*i*,f,t_) can be expressed as eqn (23)23
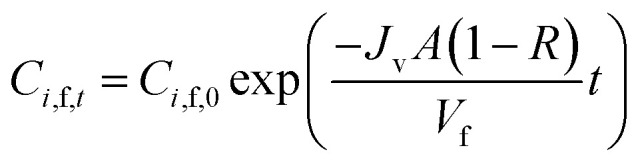


In order to evaluate the diananofiltration process, we define the remove rate of solutes (*G*_*i*_) as:24
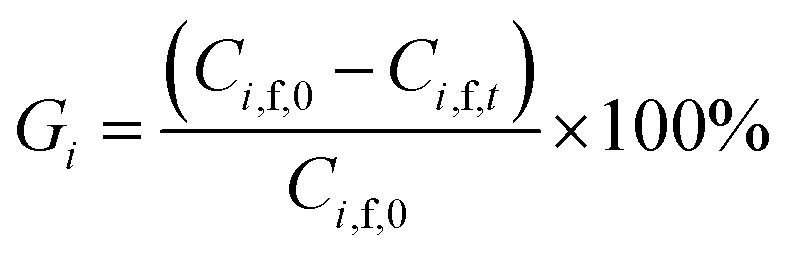


Based on the definition of *G*_*i*_, the following parameters were defined as well. They were used in the model optimization process. The total inhibitor remove rate is defined as eqn (25). The water consumption efficiency in the diananofiltration process is described as eqn (26). The inhibitors remove efficiency is described as eqn (27) The recovery rate of monosaccharides is described as eqn (28)25
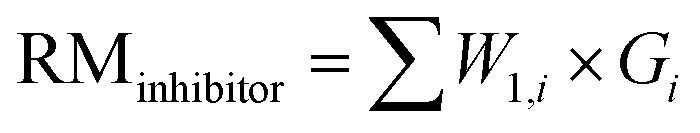
26
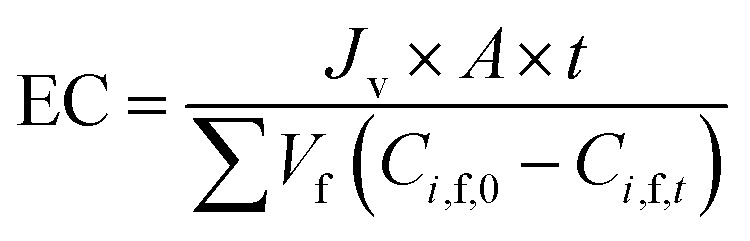
27
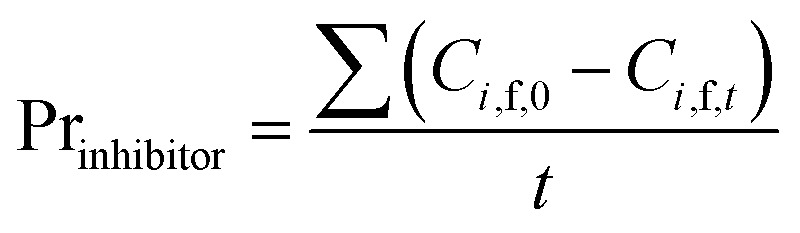
28
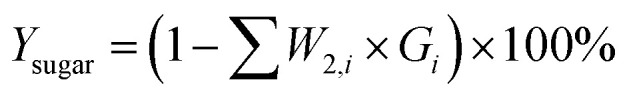
among which, *W*_1,*i*_ is the mass fraction of inhibitors. *W*_2,*i*_ is the mass fraction of monosaccharides.29
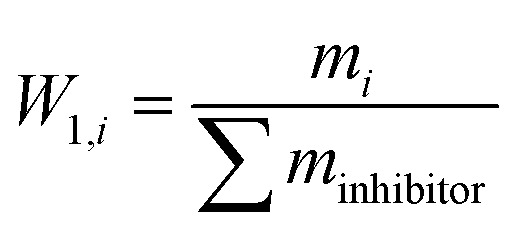
30
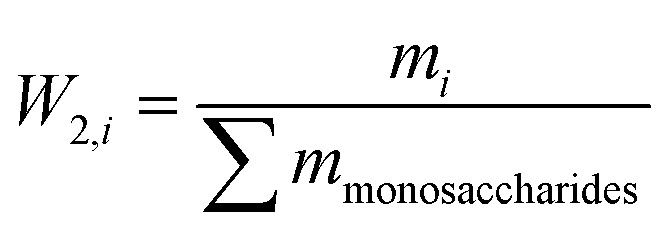


In order to evaluate the fitness of the model predictions to the experimental data, the average relative deviation (ARD%) between experimental and predicted data was calculated by the following equation:31
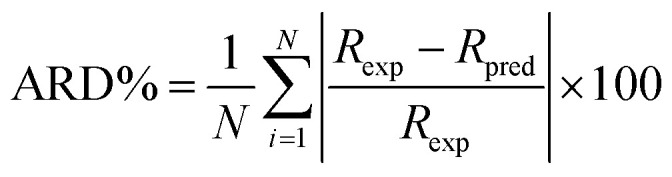
where *R*_exp_ and *R*_pred_ is the experimental and predicted rejection, respectively. *N* is the number of experiment data points.

## 4. Results and discussion

### 4.1. Calculation of the model parameters

The structural parameters of the membrane, *i.e.*, the membrane pore radius (*r*_p_) and the membrane thickness (Δ*X*/*A*_k_), have a great influence on prediction of the membrane performance, and these parameters can be obtained from physical methods, such as atomic force microscopy or scanning electron microscopy.^23^ In this context, Liu *et al.*^24^ proposed a correlation between the molecular weight cut-offs (MWCO) and the *r*_p_, and reported an *r*_p_ of approximately 0.39 nm for the DK1812 membrane. In our case, the rejection data were fit to the Spiegler–Kedem and the steric hindrance pore models to find the pore radius, as these methods were previously employed by Fang *et al.*^25^ Thus, the *r*_*p*_ of the DK membrane calculated from the model was 0.395 nm at pH 3. With the value of *r*_p_ in hand, the value of Δ*X/A*_k_ could be calculated from the Hagen–Poiseuille equation (eqn (18)), in which the pure water permeability *J*_w_/Δ*P* was approximately 2.269 × 10^−11^ m Pa^−1^ s^−1^ at pH 3, as determined by a pure water permeate experiment. Indeed, this value of *J*_w_/Δ*P* was similar to that reported by Almazán *et al.*^26^ (*i.e.*, 2.79 × 10^−11^ m Pa^−1^ s^−1^). Thus, the *r*_p_ and Δ*X*/*A*_k_ values calculated from the model were 0.395 nm and 1.661 μm, respectively, and so these values were employed in the following DSPM-DE model.

An additional membrane parameter, namely the membrane volume charge density (*X*_d_), which is essential for calculating the rejection of an ionic compound, was obtained by fitting the rejection data of a Na_2_SO_4_ solution obtained at a range of concentrations (C_b_). The relationship between *X*_d_ and *C*_b_ is defined as a form of the Freundlich isotherm^27^ as follows:32
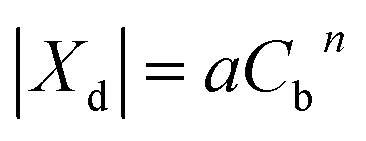


**Fig. 2:**
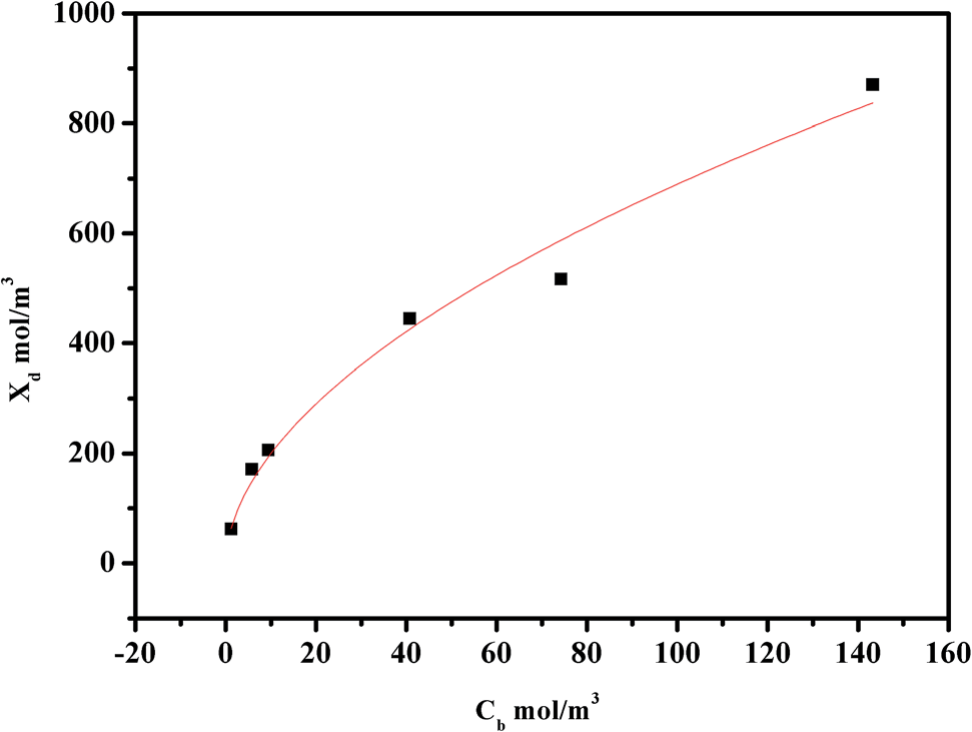
Relationship between the effective charge density *X*_d_ and the Na_2_SO_4_ concentration.

For the system of interest herein, the correlation between *X*_d_ and *C*_b_ is shown in Fig. 2, which gives *a* and *n* values of 57.94 and 0.5379, respectively. Consequently, when the concentration of Na_2_SO_4_ is known, the volume charge density *X*_d_ can be easily determined.

### 4.2. Influence of solution pH on the membrane separation performance

**Fig. 3:**
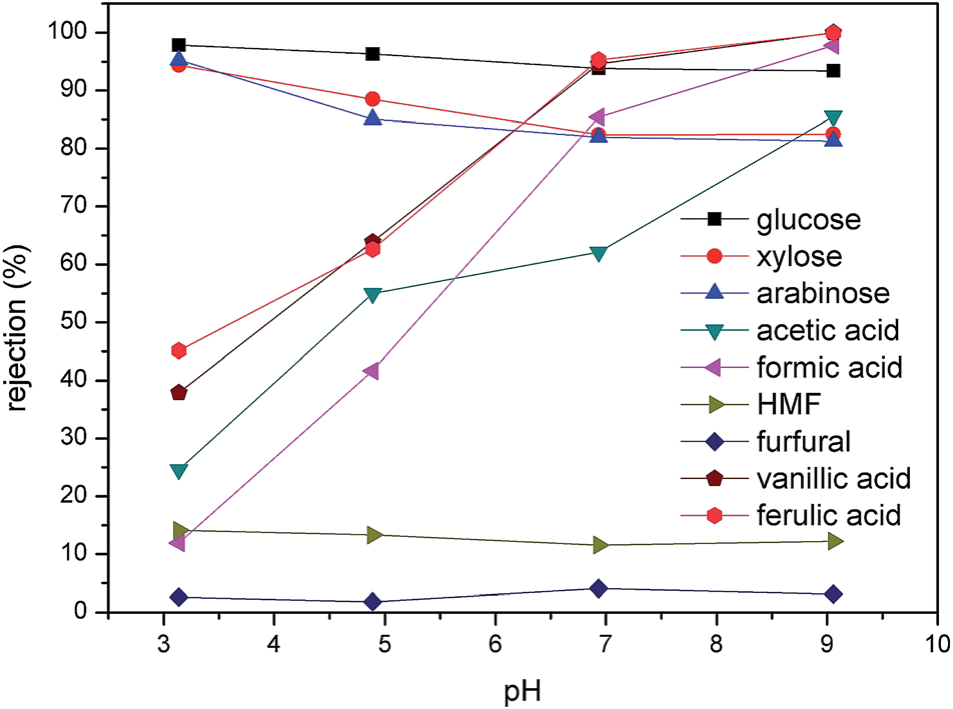
Effect of pH on the rejection of the various compounds present in the hydrolysate. An operating pressure of 20 bar was employed along with a feed temperature of 25 °C.

The molecular formula, dissociation constant, diffusion coefficient, and Stokes diameter of typical sugars, furans, phenolic compounds, and carboxylic acids present in the dilute acid corncob hydrolysate are shown in Table 1. As previously reported, the sieving mechanism and the Donnan exclusion are the two main mechanisms of molecular separation in the NF process.^28^ In the case of uncharged solutes present in the hydrolysate, such as glucose, xylose, arabinose, HMF, and furfural, their separation performances depend mainly on the sieving mechanism. Thus, the rejection percentages of these components at a range of pH values are shown in Fig. 3. More specifically, at pH 3.14, glucose exhibited the largest rejection, followed by xylose, arabinose, HMF, and furfural. This trend is in accordance with the particle sizes of the five molecules, as indicated in Table 1. Upon increasing the pH to 9.05, the rejection of glucose, xylose, and arabinose decreased from 97.84, 94.38, and 95.25%, to 93.36, 82.47, and 81.28%. Indeed, similar results were previously reported,^29,30^ it was assumed that the increase in solution pH may facilitate membrane swelling.

**Table 1:** Physical properties of the sugar and inhibitor compounds present in the corncob hydrolysate^12^

	Molecular formula	Stokes diameter (nm)	Diffusion coefficient (10^−6^cm^2^ s^−1)^	Dissociation constant
Xylose	C_5_H_10_O_5_	0.638	6.76	12.28
Glucose	C_6_H_12_O_6_	0.73	7.69	12.15
Arabinose	C_5_H_10_O_5_	0.635	7.73 (12)	12.34
Furfural	C_5_H_4_O_2_	0.412	11.2	High (>12)
HMF	C_6_H_6_O_3_	0.463	10.6	High (>12)
Acetic acid	CH_3_COOH	0.412	11.9	4.756
Formic acid	HCOOH	0.323	15.2	3.751
Ferulic acid	C_10_H_10_O_4_	0.58	8.1	4.27
Vanillic acid	C_8_H_8_O_4_	0.48 (12)	10.1	4.08

In contrast, the rejection of carboxylic acid and phenolic compounds increased significantly upon increasing the solution pH (Fig. 3). In this case, an increase in the pH from 3.14 to 6.93, resulted in the rejections of acetic acid, formic acid, vanillic acid, and ferulic acid increasing from 24.58, 11.94, 37.9, and 45.14% to 62.1, 85.4, 94.6, and 95.26%, respectively. A further increase in pH to 9.05 resulted in rejections of almost 100% for the four acids, thereby indicating that these compounds essentially did not pass through the membrane, a phenomenon also observed by Li *et al.*^31^ This large variation in the rejection of carboxylic acid and phenolic compounds at a given pH value was therefore expected to be correlated to their respective p*K*_a_ values. As shown in Table 2, the p*K*_a_ values for acetic acid, formic acid, vanillic acid, and ferulic acid are within the range of 3.5–5. Thus, upon variation in the solution pH within this region, the dissociation degrees of the acids changed dramatically. Indeed, at pH 3.14, only 1.9% of acetic acid, 16.1% of formic acid, 4.32% of vanillic acid, and 5.63% of ferulic acid are dissociated, and so the sieving mechanism dominated during the NF process. However, when the pH was increased to levels greater than the p*K*_a_ values, these compounds were essentially fully dissociated in solution. Moreover, the isoelectric point of the DK membrane (*i.e.*, a value of approximately 4)^32^ should also be considered, as it resulted in similar changes in the membrane surface charge upon varying the solution pH. At pH values higher than the membrane isoelectric point, the surface of the membrane was negatively charged. As such, the increased rejection of carboxylic acid and phenolic compounds at pH 6.93 and 9.05 was attributed to the enhanced electrostatic repulsion between the membrane and the negatively charged solute.^31^

**Table 2:** Operating parameters and separation performances of the diananofiltration process for purification of the dilute acid corncob hydrolysate at the minimum EC, maximum Pr_inhibitor_, and maximum *Y*_sugar_ values

Point	Variable		EC (L g^−1^)	Pr_inhibitor_ (g min^−1^)	*Y*_sugar_ (%)
	*J*_v_ (L m^−2^ min^−1^)	t (min)			
(P1)	0.94	34.75	0.97	0.31	83.03
(P2)	2.40	18.50	1.31	0.59	80.97
(P3)	1.10	31	1.01	0.35	83.15

### 4.3. Effect of SO_4_^2−^ concentration

**Fig. 4:**
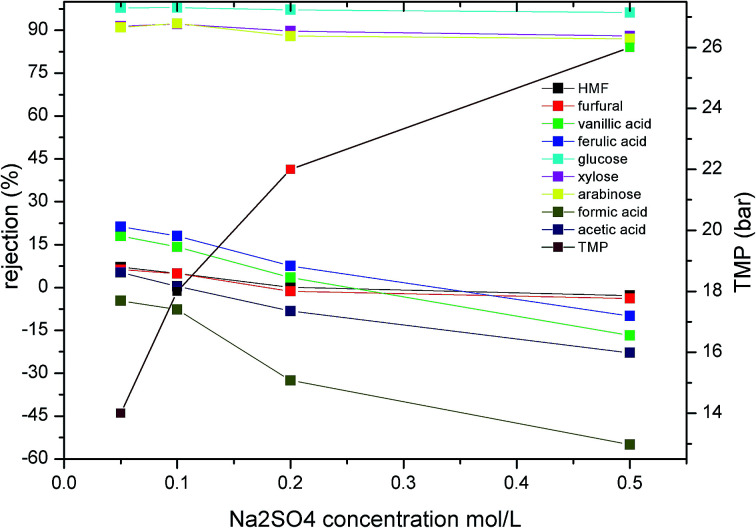
Effect of Na_2_SO_4_ concentration on the rejection of the various solutes present in the hydrolysate. A permeate flux of 1.35 L m^−2^ min^−1^ was employed along with a pH of 3 and a feed temperature of 25 °C (TMP is transmembrane pressure).

It has been widely confirmed that the inorganic salt concentration of a solution has a significant influence on the membrane separation performance during NF.^33^ As the concentration of H_2_SO_4_ is approximately 0.2 mol L^−1^ in hydrolysate solutions, the effect of SO_4_^2−^ concentration on the rejection of monosaccharides, furans, and carboxylic acids should be examined. In this case, to avoid the presence of additional hydrogen ions influencing the solution pH, we employed Na_2_SO_4_ rather than H_2_SO_4_ to vary the SO_4_^2−^ concentration (see Fig. 4). In addition, to ensure a constant permeate flux, the operating pressure was adjusted according to the increased Na_2_SO_4_ concentration. As shown in Fig. 4, upon increasing the Na_2_SO_4_ concentration in the hydrolysate from 0.05 to 0.5 mol L^−1^, the rejection of glucose, xylose, and arabinose decreased by 2, 3.5 and 4%, respectively. Interestingly, the rejection of HMF and furfural decreased significantly from 7.13 and 6.25% to −2.804 and −3.834%, respectively. This trend corresponded with previous literature reports,^33,34^ it was assumed that the decrease in rejection may be attributed to salt-induced pore swelling^35^ or reduction of the hydration layer on the pore walls.^36^

In the case of the organic acids, significant changes in rejection were observed upon increasing the concentration of Na_2_SO_4_ in the mixture from 0.05 to 0.5 mol L^−1^. More specifically, the rejection of formic acid, vanillic acid, ferulic acid, and acetic acid decreased from −4.66, 18.01, 21.25 and 5.24% to −54.96, −16.728, −10.011 and −22.83%,respectively. Weng *et al.*^10,37^ also reported a similar negative rejection of acetic acid and HMF during the NF of dilute acid rice straw hydrolysate. They assumed that this decreased rejection was attributed to interactions between the concentration polarization layer of the sugars and the inhibitors, while other reports have suggested that this phenomenon may be attributed to a combination of electrostatic screening and a reduction in steric hindrance.^38^ As Na^+^ has a smaller ionic radius and moves more rapidly in solution than SO_4_^2−^, it passes more easily through the membrane. In addition, the electrostatic repulsion between SO_4_^2−^ and the membrane is higher than those of the carboxylic acids, thereby leading to the increased rejection of SO_4_^2−^ compared to the carboxylic acids. Thus, upon increasing the Na_2_SO_4_ concentration in solution, increased quantities of organic acids pass through the membrane to maintain charge balance at the membrane outlet, thereby leading to negative retention of the carboxylic acids.

### 4.4. Modeling and optimization

#### 4.4.1 Effect of permeate flux

**Fig. 5:**
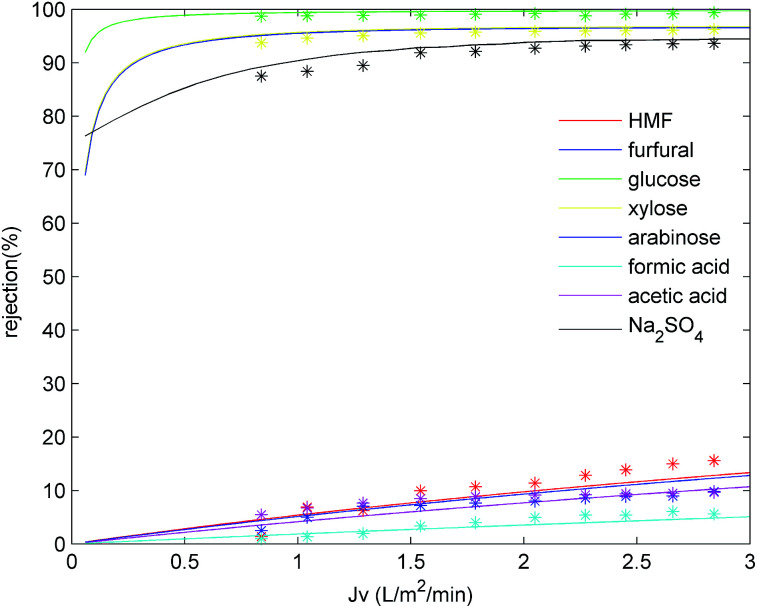
DSPM model fitting of solute rejection at different permeate fluxes. A feed pH of 3 and a feed temperature of 25 °C were employed.

Fig. 5 shows the effect of permeate flux on the rejection of the main hydrolysate components. As indicated, the rejection increased for all solutes upon increasing the permeate flux 0.84 to 2.84 L m^−2^ min^−1^, and this effect was particularly pronounced for formic acid, acetic acid, and HMF, where their rejections increased from 0.95, 5.44 and 1.38% to 5.59, 9.65 and 15.6%, respectively. Moreover, the Na_2_SO_4_ retention also increased slightly with an increase in the permeate flux. This phenomenon could be explained by the convection-diffusion mechanism.^33^ More specifically, at higher flux rates, water passes more easily through the membrane, leading to a lower solute concentration in the filtrate and higher solute retentions. However, upon further increasing the permeate flux, greater quantities of the solute accumulate at the membrane surface, thereby leading to severe concentration polarization. Thus, solute diffusion through the membrane would be enhanced, resulting in a decrease or plateau of the solute retention. This would be more likely to take place in the case of high-rejection solutes. In addition, Fig. 5 also shows the fitting data for the DSPM-DE model, where it is apparent that the DSPM-DE model fits well with the experimental data (2.15% deviation for monosaccharides, 4.6% deviation for Na_2_SO_4_). As indicated, the rejection of both monosaccharides and inhibitors increased upon increasing the permeate flux. Although an increased rejection of monosaccharides is beneficial for their recovery from the hydrolysate, the rejection of inhibitor also increased, and so it is apparent that the selection of a suitable permeate flux plays an important role in the NF process. As such, we moved on to optimize the permeate flux, as described in the following subsection.

#### 4.4.2 Multi-objective optimization of the permeate flux (*j*_v_) and operating time (*t*) during the diananofiltration process

To further improve the purities of the monosaccharides present in the retentate, a diafiltration step was introduced for inhibitor removal. Multi-objective optimization on the basis of DSPM-DE model was then carried out to select a suitable permeate flux and operating time. Three objective functions were selected, namely maximized Pr_inhibitor_ and *Y*_sugar_, and minimized EC. The permeate flux *j*_v_ and the operating time *t* are the two decision variables, where the upper value of the *j*_v_ was 2.4 L m^−2^ min^−1^ and the diananofiltration time was limited to 40 min. A total inhibitor removal rate (RM_inhibitor_) of ≥90% was set as the constraint for the purification of monosaccharides. To reduce the search region and prevent the generation of unrealistic results, a total monosaccharide removal rate (RM_monosaccharides_) of ≤35% was set as an additional constraint.

**Fig. 6:**
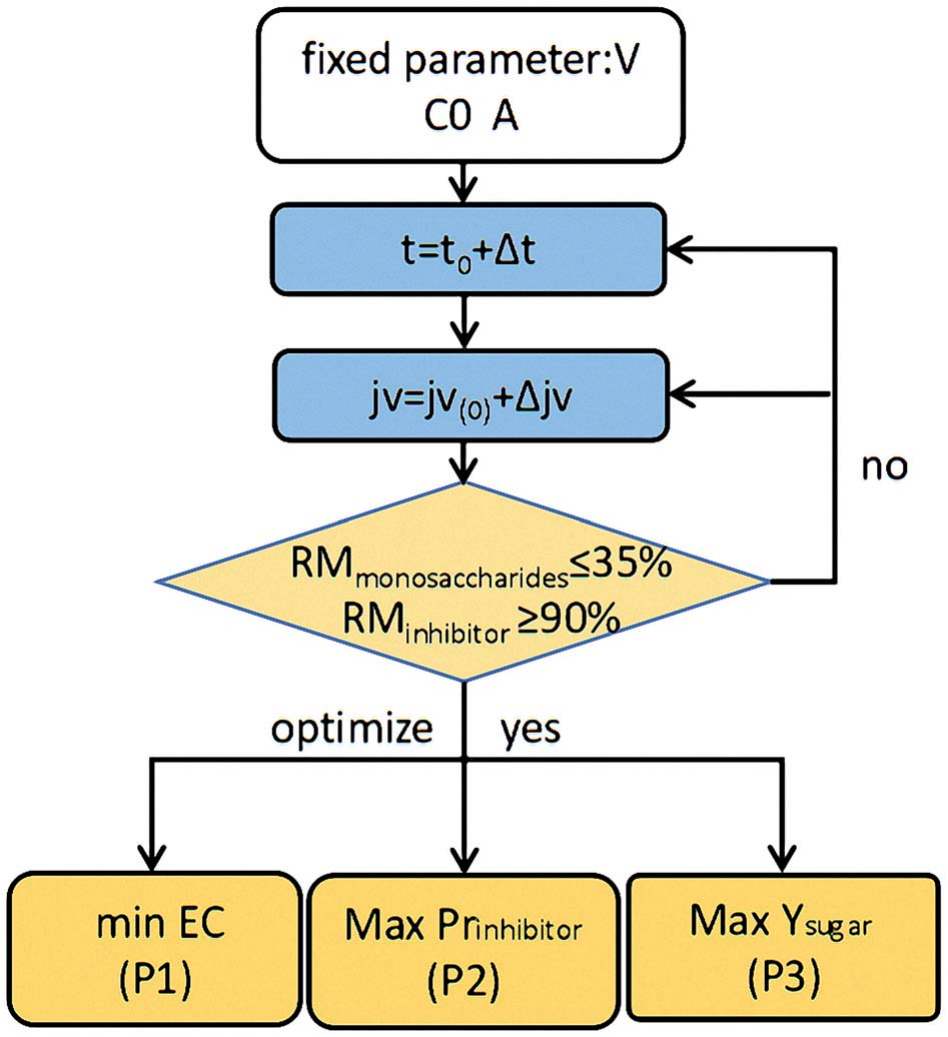
Optimization flow chart.

A parallel optimization strategy was proposed for this multi-objective optimization study. The optimization flow chart is shown in Fig. 6. More specifically, the decision variables were discrete firstly. By systematically scanning a dense grid of variable values (10 000), approximately 3320 values were then found to fulfill the set constraints. Among these values the individual operating conditions were finally obtained corresponding to the maximum *Y*_sugar_, maximum Pr_inhibitor_, and minimum EC values (*i.e.*, P3, P2, and P1 in Fig. 6). The optimization process was carried out using MATLAB Software (MathWorks).

**Fig. 7:**
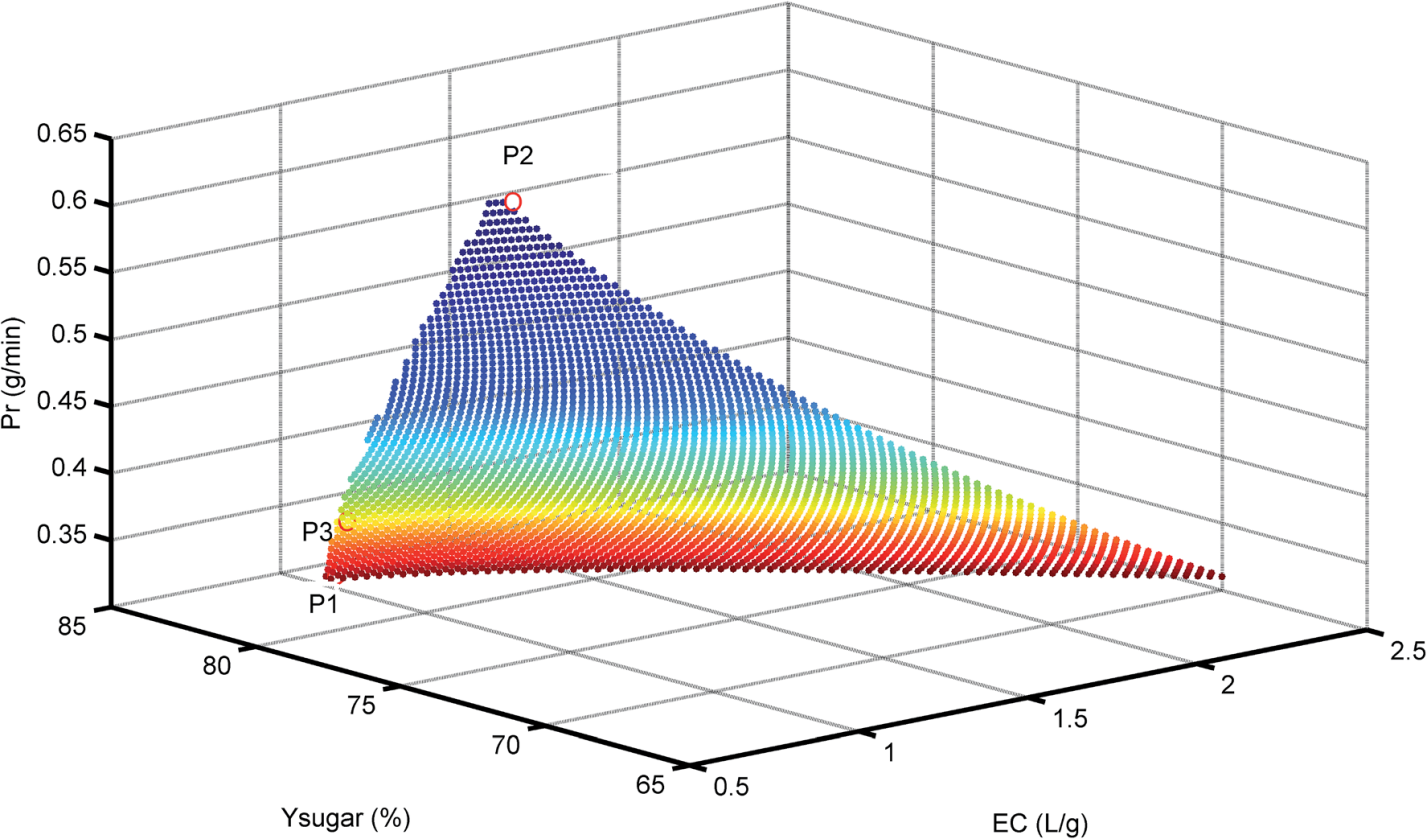
3D plot for Pr at different *Y*_sugar_ and EC values. P1 = minimum EC, P2 = maximum Pr_inhibitor_, and P3 = maximum *Y*_sugar_.

**Fig. 8:**
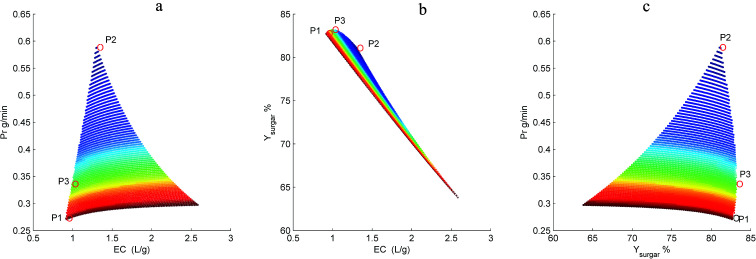
Dependence of Pr_inhibitor_ on *EC* (a), *Y*_sugar_ on EC (b), and Pr_inhibitor_ on *Y*_sugar_ (c) in the diananofiltration fractionation of the dilute acid corncob hydrolysate. P1 = minimum EC, P2 = maximum Pr_inhibitor_, and P3 = maximum *Y*_sugar_.

Fig. 7 shows the resulting 3D plots in which all the points can fulfill the design constraints, but no points exist which can meet all the optimization objectives simultaneously. For a better view, the projection planes are presented in Fig. 8. As can be seen in Fig. 8a, the Pr_inhibitor_ increases with an increase of EC. This is because increasing the water consumption results in greater quantities of inhibitors passing through the membrane. The maximum Pr_inhibitor_ value locates in point P2, while P1 and P3 have similar Pr_inhibitor_ values. Similarly, Fig. 8c shows the correlation between Pr_inhibitor_ and *Y*_sugar_. With the increase of *Y*_sugar_, the Pr_inhibitor_ increases first and then decreases. The maximum of *Y*_sugar_ lies in the point P3. The *Y*_sugar_ depends strongly on the water consumption EC (see Fig. 8b). Increasing the EC the *Y*_sugar_ decreases. This is due to the fact that water enhances the permeation of the monosaccharide into the filtrate. Thus, to increase the recovery rate of monosaccharide, the EC should be low, and at the same time the operating time should be short, and the permeate flux should be small as well. The minimum EC lies in the point P1. However, the minimum EC (P1) and maximum *Y*_sugar_ (P3) points are very close. To analyze the optimization results, we can conclude that the objective functions in terms of Pr_inhibitor_, *Y*_sugar_ and EC contradict one another. The optimal operating conditions were actually non-existed. In such case, only suitable operating conditions can be selected among the P1, P2, and P3 points. Table 2 compares the Pr_inhibitor_, *Y*_sugar_ and EC values of the points P1, P2, and P3. It can be observed P1 and P3 give the similar performance. The aim of this work is to obtain the monosaccharide as much as possible. Hence, the operating conditions of P3 were selected as the optimal conditions which would be verified use of real hydrolysate solution as the feed.

#### 4.4.3 Verification of the optimized result

**Fig. 9:**
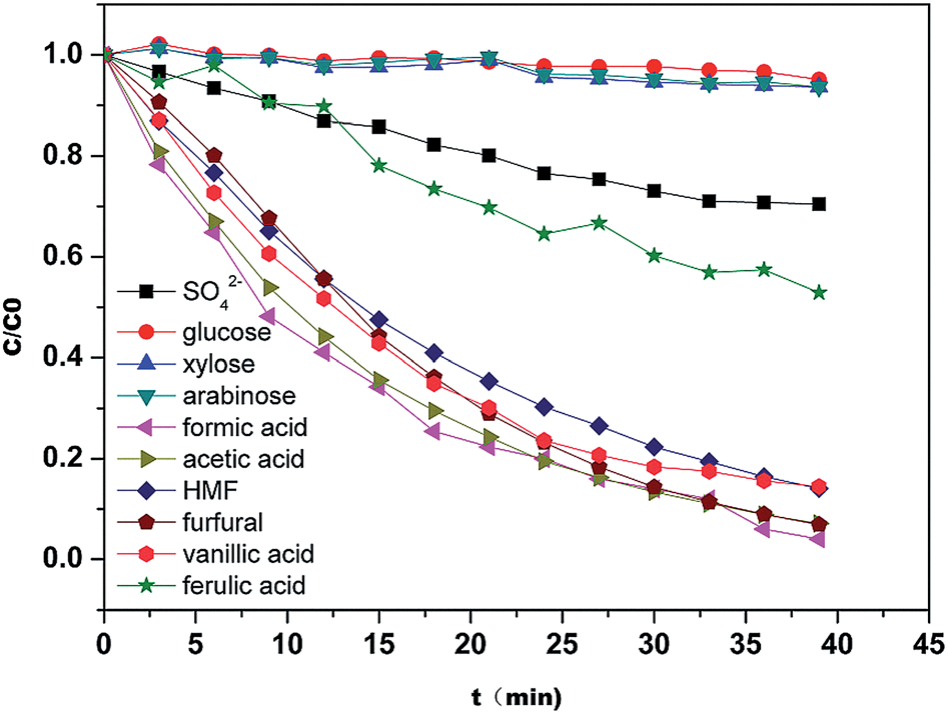
Concentration profiles of the solutes present in the hydrolysate during the diananofiltration process. The feed temperature was maintained at 25 °C.

To verify the optimized result, the diananofiltration mode was used to process the real hydrolysate solution which have a lower pH (pH < 1). The concentration profiles are shown in Fig. 9. Due to the complexity of the hydrolysate solution, the rejection for each solute was higher than the model predictions. The operating time of 35 min and 13 L of water were required to reach the target values. However, the monosaccharide loss was lower than the predicted value, with only 6.45% glucose, 9.25% xylose, and 9.47% arabinose. The deviations of approximately 4% were calculated for arabinose, glucose, H_2_SO_4_, formic acid, HMF, and furfural, while the deviation of acetic acid was >7%. After filtration 0.78 g L^−1^ acetic acid was detected in the retentate, the furan concentration was also reduced to 0.08 g L^−1^, and the total monosaccharide concentration in the retentate was in the range of 85–90 g L^−1^. These results showed that the change of solution pH might have no significant influence on the separation of monosaccharides and inhibitors when pH < 3. We could therefore conclude that the optimized operating conditions were feasible to deal with the hydrolysate solution. The remaining sulfuric acid (around 72%) has to be separated from monosaccharide by the following electrolyte exclusion chromatography.

### 4.5. Monosaccharide and sulfuric acid recovery by electrolyte exclusion chromatography

**Fig. 10:**
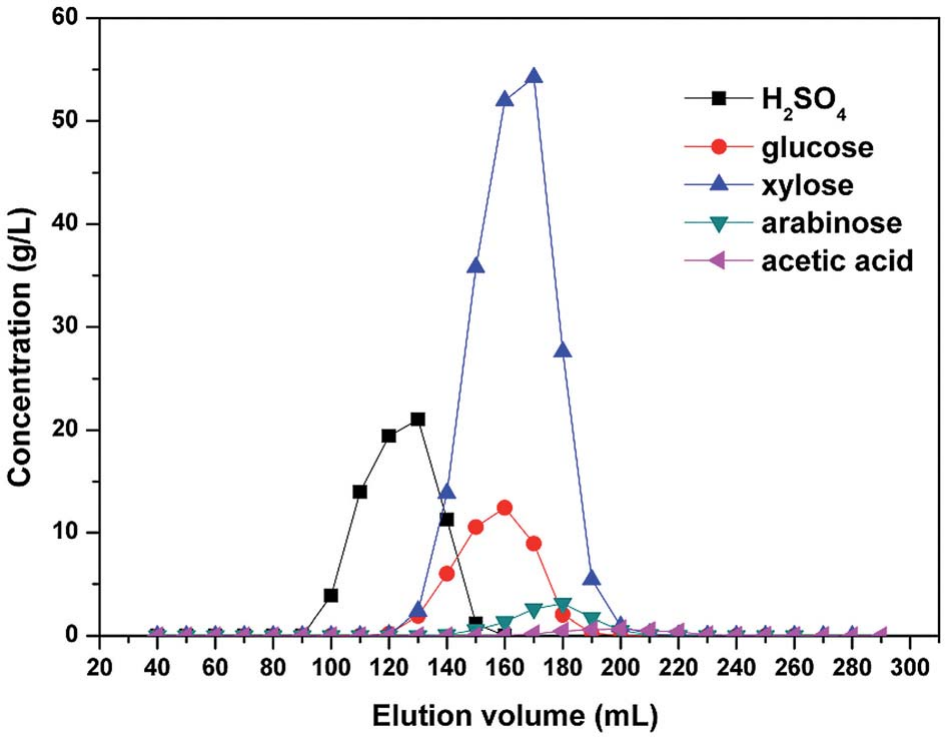
Elution chromatogram of the hydrolysates pretreated by nanofiltration. The feed temperature was maintained at 25 °C and an elution flow rate of 1 mL min^−1^ was employed.

The chromatographic recovery of both sulfuric acid and the monosaccharides from the hydrolysates pretreated by nanofiltration was then examined using batch column experiments, and an elution chromatogram of the hydrolysate obtained using a strong acid cation-exchange resin is shown in Fig. 10. As indicated, sulfuric acid was eluted first, with the breakthrough point of the sulfuric acid peak being close to the void volume of the resin bed. Subsequently, all monosaccharides were eluted simultaneously due to their similar structures, and this was followed by the elution of acetic acid. Formic acid, HMF, furfural, and the phenolic compounds were not considered here due to their low concentrations in the diananofiltrated hydrolysate.

Following pretreatment of the hydrolysates by nanofiltration, the sulfuric acid concentration was reduced to 0.3 mol L^−1^, thereby indicating that the electrolyte exclusion is sufficiently strong to prevent the SO_4_^2−^ ions from entering the resin pores. In addition, due to the electroneutrality of the solution, cations were also unable to enter the pores, thereby resulting in the poor adsorption of sulfuric acid onto the resin and consequently, its rapid elution. It should also be noted that some overlap was observed between elution of the sulfuric acid and the monosaccharides, in addition to between the monosaccharides and the acetic acid. Following the recovery of 90.37% sulfuric acid (98% pure), the overall monosaccharide yield and purity were 94.87% and 95.6–98.5%, respectively. We therefore expect that to achieve a further increase in the yields and purities of the monosaccharides and the sulfuric acid, a continuous chromatography process would be required.

## 5. Conclusions

We herein described the successful application of a combined membrane-chromatography process for the removal of inhibitors and the recycling of monosaccharides and sulfuric acid from dilute acid corncob hydrolysates. Initially, we investigated the effect feed pH, permeate flux, and Na_2_SO_4_ concentration on the retention/rejection of monosaccharides and inhibitors in a model solution to obtain the optimal conditions for the nanofiltration process. More specifically, we found that optimal separation of the carboxylic acids and furans from the monosaccharides was achieved at pH 3, while carboxylic acid and furan rejection decreased upon increasing the Na_2_SO_4_ concentration. In addition, coupling of the Donnan steric pore and dielectric exclusion model with mass balance measurements was successful both in predicting solute rejection at different permeate fluxes and in simulating the diananofiltration process. Furthermore, to determine a suitable permeate flux and operating time for the process, multi-objective optimization was carried out to obtain the maximum total inhibitor removal efficiency, the maximum recovery rate of monosaccharides, and the minimum water consumption. Indeed, the operating conditions that maximized the monosaccharide recovery rate were optimal. Subsequently, a cheap strong acid cation-exchange resin (PS-DVB) was employed to recover both the monosaccharides and the sulfuric acid from the nanofiltered hydrolysate, with elution and column regeneration being facile using water as the eluent. The suitability of the optimized operating conditions was then confirmed using hydrolysate solutions, with nanofiltration resulting in the removal of 90% of inhibitors, including HMF, furfural, phenolics, and carboxylic acids, in addition to the recovery of 93.55% glucose, 90.75% xylose, and 90.53% arabinose following treatment using a batch column packed with the strong acid cation-exchange resin. Over the whole combined process, monosaccharide losses ranged from 10 to 15%, and the recovery of dilute sulfuric acid ranged from 65 to 70%. The recovered sulfuric acid was then added directly to the subsequent hydrolysis process, while the monosaccharides were continuously supplemented to the fermenter. As such, our results clearly demonstrated that the combination of nanofiltration with electrolyte exclusion chromatography is an effective strategy for the removal of inhibitors and the recovery of monosaccharides from dilute acid corncob hydrolysates.

## Conflicts of interest

The authors declare there is no conflicts of interest regarding the publication of this paper.

## Abbreviation

*A*_m_
: Membrane area m^2^
*A*_k_
: Porosity of the membrane%
*c*_*i*_
: Concentration of *i*th component within pore mol L^−1^
*C*_*i*,f_
: Solutes concentration of feed mol L^−1^
*C*_*i*,f,*t*_
: Concentration of solutes at *t* mol L^−1^
*C*_*i*,*x* = 0_
: Concentration of *i*th component in membrane surface adjacent to the feed solution mol L^−1^
*C*_*i*,*x* = Δ*X*_
: Concentration of *i*th component in membrane surface adjacent to the permeate solution mol L^−1^
*C*_*i*,m_
: Concentration of *i*th component in feed solution near to membrane surface mol L^−1^
*C*_*i*,p_
: Concentration of *i*th component in permeate solution mol L^−1^
*C*_*i*,b_
: Bulk concentration of *i*th component in feed solution mol m^−3^
*d*
: Thickness of oriented solvent layer (0.28 nm) m
*d*_c_
: Hydrodynamic diameter cm
*D*_*i*_
: Effective diffusivity of *i*th component m^−2^ s^−1^
EC: Water consumption respect to inhibitors L g^−1^
*F*
: Faraday constant, 96 487 C mol^−1^
*G*_*i*_
: Remove rate of solute i%
*j*_*i*_
: Flux of *i*th component mol m^−2^ s^−1^
*J*_v_
: Volumetric permeation flux m^3^ m^−2^ s^−1^
*J*_w_
: Water flux m^3^ m^−2^ s^−1^
*k*
: Mass transfer coefficient m s^−1^
*K*_*i*,c_
: Hindrance factor for convection dimensionless
*K*_*i*,d_
: Hindrance factor for diffusion dimensionless
m_*i*_: Mass concentration of solute g L^−1^
*m*_inhibitor_
: Mass concentration of inhibitor g L^−1^
*M*_monosaccharides_
: Mass concentration of monosaccharides g L^−1^
Pe: Peclet number dimensionless
Δ*P*: Applied transmembrane pressure bar
Pr_inhibitor_: inhibitors remove efficiency g min^−1^
*r*_*i*,s_
: Stokes radius of *i*th component nm
*r*_p_
: Average membrane pore radius nm
*R*
: Universal gas contant 8.314 J mol^−1^ K
*R*_*i*,real_
: Real rejection of *i*th component%
*R*_*i*,obs_
: Observed rejection of *i*th component%
*R*_exp_
: Predicted rejection%
*R*_pred_
: Experimental rejection%
Re: Reynolds number dimensionless
RM_inhibitor_: Total inhibitor remove rate%
RM_monosaccharides_: Total monosaccharides remove rate%
Sc: Schmidt number dimensionless
Sh: Sherwood number dimensionless
*t*
: Operate time min
*T*
: Absolute temperature K
*V*_f_
: Volume of feed solution L
*W*_1,*i*_
: Mass fraction of inhibitors%
*W*_2,*i*_
: Mass fraction of monosaccharides%
*x*
: Axial position within the membrane cm
*Y*_sugar_
: Total monosaccharides recovery rate%
*X*_d_
: Effective membrane volume charge density mol m^−3^
*z*_*i*_
: Valence of ion i
*μ*
: Viscosity of solution Pa*s
*Ψ*
: Electrostatic potential *V*
*λ*_*i*_
: Ratio of stokes radius for solute i and membrane pore radius dimensionless
*ϕ*_*i*_
: Equilibrium partition coefficient dimensionless
*ρ*
: Density of solution g cm^−3^
ε*: Dielectric constant of oriented water layer (ε* = 6) dimensionless
*ε*_b_
: Bulk dielectric constant (*ε*_b_ = 80) dimensionless
*ε*_p_
: Pore dielectric constant dimensionless
*ε*_0_
: Permittivity of free space (8.85419 × 10^–12^ C J^−1^ m^−1^)
Δ*W*_*i*_: Born solvation energy barrier J

## Supplementary Material
